# Distribution and Pathogenicity Differentiation of Physiological Races of *Verticillium dahliae* from Cotton Stems in Western China

**DOI:** 10.3390/pathogens13070525

**Published:** 2024-06-21

**Authors:** Jianwei Zhang, Aerguli Jiamahate, Hui Feng, Tohir A. Bozorov, Dawei Zhang, Jianwei Guo, Honglan Yang, Daoyuan Zhang

**Affiliations:** 1 College of Agronomy, Xinjiang Agricultural University, Urumqi 830052, China; zjw18294830794@163.com; 2 State Key Laboratory of Desert and Oasis Ecology, Key Laboratory of Ecological Safety and Sustainable Development in Arid Lands, Xinjiang Institute of Ecology and Geography, Chinese Academy of Sciences, Urumqi 830011, China; 18040921335@163.com (A.J.); tohirbozorov@yahoo.com (T.A.B.); 3 State Key Laboratory of Biocontrol and Guangdong, Provincial Key Laboratory of Plant Resources, School of Life Sciences, Sun Yat-sen University, 135 Xingang West Road, Guangzhou 510275, China; fengh35@mail2.sysu.edu.cn; 4 Laboratory of Molecular and Biochemical Genetics, Institute of Genetics and Plants Experimental Biology, Uzbek Academy of Sciences, Yukori-Yuz, Kibray 111226, Uzbekistan; 5 Research Institute of Economic Crops, Xinjiang Academy of Agricultural Sciences, Urumqi 830091, China; zbzdw012@126.com; 6 College of Agronomy and Life Sciences, Yunnan Urban Agricultural Engineering and Technological Research Center, Kunming University, Kunming 500600, China; gjwkf475001@sina.com; 7 Turpan Eremophytes Botanical Garden, Chinese Academy of Sciences, Turpan 838008, China

**Keywords:** physiologic races, pathogenicity, *Verticillium* wilt, *V. dahliae* identification

## Abstract

Verticillium wilt, caused by the pathogenic fungus *Verticillium dahliae*, has emerged as a severe threat to cotton globally. However, little is known about the genetic diversity of this pathogen in an infected single cotton plant. In this study, we isolated three new *V. dahliae* strains from the disease stems of *Gossypium hirsutum* from the cotton field in Western China and assessed their pathogenicity to the cotton cultivar Xinnongmian-1 and its two transgenic lines, as well as two laboratory strains, VD592 and VD991. These three new *V. dahliae* strains were identified using DNA barcodes of tryptophan synthase (TS), actin (ACT), elongation factor 1-α (EF), and glyceraldehyde-3-phosphate dehydrogenase (GPD). Moreover, the haplotype analysis revealed that the three new races had distinct haplotypes at the TS locus. Furthermore, the results of culture features and genetic diversity of ISSR (inter-simple sequence repeat) revealed that there were separate *V. dahliae* strains, which were strong defoliating pathotypes belonging to race 2 type, as determined by particular DNA marker recognition. The identified strains demonstrated varied levels of pathogenicity by leaf disc and entire plant inoculation methods. Conservatively, these strains showed some pathogenicity on cotton lines, but were less pathogenic than the reference strains. The findings revealed that several strong defoliating *V. dahliae* pathotypes coexist on the same cotton plant. It indicats the importance of regular monitoring as an early warning system, as well as the detection and reporting of virulent pathogen strains and their effects on crop response.

## 1. Introduction

The genus *Verticillium* sensu stricto (Ascomycota, Sordariomycetes, Plectosphaerellaceae), a well-known class of ascomycete filamentous fungi that includes many phytopathogenic fungi, has caused significant economic losses globally [1]. The taxonomic status of the *Verticillium* genus presents challenges due to the genetic diversity within its pathogen populations; this diversity can lead to variations in pathogenicity and host plants [2,3,4]. The taxonomic status of the genus, which includes ten species of plant pathogenic fungi, including *V. albo-atrum*, *V. alfalfae*, *V. dahliae*, *V. isaacii, V. klebahnii*, *V. longisporum*, *V. nonalfalfae*, *V. nubilum*, *V. tricorpus*, and *V. zaregamsianum*, was finalized in 2011 after rigorous taxonomic research and analysis [5]. The pathogenicity of *Verticillium dahliae* can vary significantly, and it is often assessed using various indices, including wilting type (deciduous and non-deciduous) and pathogenicity degree (strong and weak) [6].

Verticillium wilt is characterized by wilting and yellowing of leaves, vascular bundle browning and, ultimately, premature death primarily caused by *V. dahliae* Kleb. [7]. *Verticillium dahliae* is a cosmopolitan group of ascomycete fungi, and its genetic adaptability allows it to infect a broad spectrum of plant species ranging from annual or perennial crops, fruit, ornamental trees, shrubs, or herbaceous plants [8]. As outbreaks of Verticillium wilt have occurred on new hosts, the number of hosts continues to expand, indicating that this destructive plant disease pathogen poses a threat to crop production and forest health globally [9]. The mechanism of invasion by *Verticillium dahliae* is well understood and typically involves several stages mainly through small hyphae germinating to produce mycelium and attaching to the root surface, invading the root, proliferating, and entering the plant xylem [4,10]. Cotton is an important oilseed and fiber crop that is very sensitive to *Verticillium* infection, which significantly affects its fiber quality and productivity and is considered the “cancer” of cotton [11].

*Verticillium dahliae* has a complicated population structure that includes deciduous and non-deciduous types, as well as various nutritional affinity groups, and mating types [6]. In the past, the identification of *Verticillium* species often relied on the characterization of their resting structures, which typically include resting mycelium, chlamydospores, and microsclerotia, which can vary in morphology and size between different species and strains [12]. While traditional morphological and physiological features, such as resting structures and mycelial pigmentation, have historically been used for the identification of Verticillium wilt pathogens, studies have shown these evidences may not be sufficient to classification [13]. Therefore, the development of molecular identification methods has been crucial in addressing the challenges associated with traditional morphological identification of *Verticillium* species. The use of polymerase chain reaction (PCR) and specific primers targeting the internal transcribed spacer region (*ITS*) of the fungal genome has revolutionized the field of fungal taxonomy and diagnostics. The PCR-based assay successfully differentiated between different species, thus opening up a new way to discriminate among *V. albo-atrum*, *V. dahliae*, and *V. tricorpus*. Several genes, including tryptophan synthase, actin (ACT) [14], elongation factor 1-α (EF*1-α*) [15], glyceraldehyde-3-phosphate dehydrogenase (GPD), and others [5], have been explored as potential DNA barcodes for fungal identification. Since then, the molecular characterization of *Verticillium*. spp has been initiated [14,16,17,18].

Aside from the molecular characteristics and genetic markers, there are several features that can be used to identify *V. dahliae*. First, *V. dahliae* were classified into two groups based on the virulence phenotype after cotton infection: defoliating (D) and non-defoliating (ND) types [19]. The D pathotype is distinguished by its propensity to induce early leaf drop, whereas the ND pathotype is characterized by causing leaf chlorosis and necrosis without premature leaf loss. This classification is crucial for understanding the specific impacts of these pathotypes on infected plants. Moreover, the severity of pathogenicity in *V. dahliae* can be stratified into strong, moderate, or weak strains, determined by the level of host morbidity and disease indices. Second, *V. dahliae* can be differentiated into three types based on their growth stages—hyphae, sclerotia, and intermediate types—which is vital for comprehending the biology and behavior of this pathogen [20]. These types display varying degrees of adaptability and resilience to different environmental conditions. Notably, this fungus can persist in the soil as microsclerotia for over a decade [18,19]. Upon sensing host root exudates, these microsclerotia germinate hyphae capable of penetrating the root cortex and colonizing the xylem vessels. Additionally, the capacity for pathogens to mutate and diversify within a population can result in the evolution of distinct pathotypes within a species. These pathotypes may have the potential to either circumvent host resistance mechanisms or exhibit varied responses to environmental stimuli. Consequently, *Verticillium* disease poses a significant challenge to control, given the complexity of its pathogenic mechanisms [21]. Third, *V. dahliae* displays different pathogenic races, identified by the presence or absence of specific virulence factors, classified as physiological races 1 and 2, respectively. A key factor differentiating these races is the *Ave1* gene; strains harboring the *Ave1* gene can trigger a defense response in certain host plants by activating the *Ve1* resistance gene [22]. Race 2 is generally more virulent than race 1 due to the absence of the *Ave1* gene, which means it cannot trigger the disease resistance conferred by the *Ve1* gene in host plants [19]. Race 2 is prevalent and can cause disease in a variety of cultivars [17,23,24]. Advanced molecular biotechnology techniques, including Restriction Fragment Length Polymorphism (RFLP), Random Amplified Polymorphic DNA (RAPD), and Inter-Simple Sequence Repeat (ISSR) analysis, have proven invaluable in assessing the genetic diversity of *V. dahliae*. Integrating these modern genetic engineering techniques with traditional breeding methods can markedly expedite the development of cotton cultivars resistant to *Verticillium* wilt.

Little is known about characterizing the pathogenicity and phylogenetic diversity of *V. dahliae* races, which is a critical area of research. To clarify the characteristics of *V. dahliae* in a single cotton plant, pathogen strains were isolated and purified from diseased Xinnongmian-1 cotton cultivar. The four universal DNA barcodes (*ACT*, *GPD*, *EF*, and *TS*) were used to distinguish the species. Subsequently, the cultural characterization of phenotypic characteristics, the rigorous determination of physiologic pathotypes and assessment of genetic diversity using ISSR markers, provided a comprehensive approach to understanding the biology and diversity of *V. dahliae* starins. Further, to ascertain the generalization of pathogenicity in cotton, selecting two resistance breeding cotton lines along with its susceptible Xinnongmian-1 cultivar, we assessed the virulence of pathogen strains. Employing both leaf disc and whole plant inoculation methods, pathogenicity analysis was carried out. Our findings obtained from the comprehensive analysis of fungi pathogenicity, genetic diversity assessment using ISSR markers, and characterization of phenotypic traits in cotton lines will serve as valuable references for various aspects of cotton breeding for tolerance. Our findings will provide references for *V. dahliae* resistance, cotton introduction, and offspring-resistant cotton breeding.

## 2. Materials and Method

### 2.1. Plant Materials and V. dahliae Strains

To dissect pathogenicity of *V. dahliae*, strains of *G. hirsutum* cultivar ‘Xinnongmian-1’ and its two independently offspring-resistant cotton lines (L16 and L38) were grown under greenhouse conditions (28 ± 2 °C/30 ± 5% RH) [25]. The *V. dahliae* reference strains VD991 and VD592 were used as controls that were kindly provided by Prof. Zhao Huixin, Xinjiang Normal University, and by Prof. Guo Huishan, Institute of Microbiology, Chinese Academy of Sciences, respectively. The three new strains were isolated from infected cotton stems of Xinnongmian-1 cultivar planted in the Manas Agricultural Experimental Station of the Xinjiang Academy of Agricultural Sciences (44°18′13.91″ N, 86°13′11.03″ E; 456 m asl).

### 2.2. Isolation, Identification, and Characterization of V. dahliae Isolates

*Verticillium*-infected cotton (cv. Xinnongmian-1) stems were cut into sections and then divided into two to six pieces for surface sterilization. Surface sterilization was held for 3 min by immersing in 10% household bleach (40 g L^−1^ of active chlorine), followed by a 3 min rinse in sterile water and air dried under a laminar flow cabinet. Stem segments ranging in length from 2 to 4 mm were aseptically transferred onto petri plates containing potato dextrose agar (PDA) (PDA, Rishui Company, Qingdao, China). Plates were incubated at 25 °C for 72 h in a thermostat incubator.

To measure the fungi growth rate and sporulation rate in vitro, we selected Czapek Dox and Potato Dextrose Broth (PDB) as the basic media [26]. The nutrient mixture of Czapek Dox consisted of sucrose (30 g/L), di-potassium phosphate (1 g/L), magnesium sulfate (0.5 g/L), potassium chloride (0.5 g/L), and ferrous sulfate (0.01 g/L), with pH 5.5, when the PDB medium consisted of 4 g per liter of potato peptone and 20 g per liter of glucose with pH 6.0. Fungi grown on PDB plates for 10 days at 25 °C were punched out of a 0.5 cm diameter solid fungal piece from the perimeter of the fungi colony using a hole puncher and placed onto the middle of the fresh PDA plate, and then plates were cultured for 15 days at 25 °C. The growth rates were subsequently calculated based on the colony size. Through the measurement of the colony size in four directions around the colony, the calculation of radial hyphal growth rate was conducted as a daily average. Experiments were repeated five times. 

The colonies were morphologically observed under the microscope and the stereoscope (Olympus, Tokyo, Japan). To isolate and validate the *Verticillium* clones, complete genomic DNA was extracted from every single purified fungal colony (Beijing Solarbio Science & Technology Co., Ltd., Beijing, China). Molecular identification was carried out using four primer pairs *ACT*, *GPD*, *EF*, and *TS* (Table S1). The PCR reaction was 20 µL, containing 10 µL 2 × EasyTaq, 1 µL of each primer (forward and reverse, 25 pmol/L^−1^), 25 ng of template DNA, and double distilled water. The PCR reaction program was as follows: 94 °C for 5 min; 94 °C for 30 s, 55 °C for 30 s, 72 °C for 30 s, 30 cycles, and finally 72 °C for 10 min. The PCR products were sequenced by Sangon Biotech Company (China). Similarity searches were performed using the Basic Local Alignment Search Tool (BLAST) by comparing the consensus sequences with those in the NCBI GenBank database (www.ncbi.nlm.nih.gov, accessed on 7 April 2024). The sequences of each locus of *V. dahliae* with high homology in the results were obtained, which were matched and aligned using MAFFT software. Further, the phylogenetic trees were constructed using IQtree based on maximum likelihood (ML) estimation.

Based on the morphological characteristics and barcode molecular results, we selected three unique isolates (named VD1, VD2, and VD3) for further study. The isolates were stored as a microconidial suspension in 25% glycerol stock at −80 °C for further use.

### 2.3. Genetic Diversity of Verticillium Isolates

Analyzing genetic diversity via inter-simple sequence repeat (ISSR) using UBC Primer Set #9 (Microsatellite) (Table S1) was performed. Amplified PCR products were separated by agarose electrophoresis in 3% agarose gels and visualized with a UV light transilluminator, and compared with two different DNA ladders of various sizes (100–2000 bp; 100–1000 bp) (Takara, Dalian, China). A binary matrix was conducted based on the presence (1) or absence (0) of the PCR product for analysis of genetic diversity. The genetic similarity matrices are obtained by the Jaccard similarity coefficient. NTsys 2.10e software was utilized for analysis, with the similarity package using a qualitative similarity coefficient (SM). Cluster analysis using the unweighted paired group method with arithmetic averages (UPGMA) algorithm was applied. The results are plotted using the tree plots in the Graphic package.

To clarify the physiological pathogenic type of fungal strains and determine whether they belong to the cotton defoliation type or not, as well as to identify their race, specific molecular markers designed for *Verticillium* were used. Total genomic DNA from each *Verticillium* isolate was extracted using the Fungi Genomic Extraction Minikit (Solarbio Biotechnology, Cat. D2300, Beijing, China), following the manufacturer’s instructions. The quality and concentration of DNA samples were determined using a NanoDrop 2000 (Thermo Fisher Scientific, Waltham, USA). DNA samples were adjusted to a final concentration of 10 ng µL^−1^ and stored at −20 °C for further use. The molecular characterization of all the isolates was performed using race and pathotype-specific diagnostic primers described for *V. dahliae* [19,24,27]. Diagnostic primer pairs VdAve1F/VdAve1R and VdR2F/VdR2R were used to determine race 1 and/or race 2 (Table S1). Determination of D and ND fungal pathotypes was ascertained using primer pairs D1/D2, INTD2F/INTD2R, ND1/ND2, and INTNDF/INTNDR (Table S1). Optimization for PCR reaction was carried out in a final volume of 20 µL containing 10 µL of 2 × PCR mix, 1.0 µL of template DNA, 1 µL of each 10 µmol/L primer (F + R), and 9.0 µL of double distilled water. Amplification conditions were as follows: 4 min denaturation at 94 °C, followed by 35 cycles of 1 min denaturation at 94 °C, 1 min of appropriate annealing temperature, 1 min of extension at 72 °C, and a final extension step of 10 min at 72 °C. All reactions were completed in a thermocycler (Bio-Rad, USA). Performing haplotype analysis with DnaSP [28] and constructing haplotype networks using PopArt version 1.7 software [29] with the median-joining method to show variation between the varied haplotypes in physiologic races of *V. dahliae* using *TS* sequences of three isolates, two reference strains, and eighty-eight homologs retrieved from NCBI.

### 2.4. Pathogenicity Assays for Isolates

The pathogenicity of fungal isolates was determined using leaf disc and entire plant inoculation techniques. For the leaf inoculation studies, healthy leaves of uniform size were cut from the petiole and washed three times with 75% ethanol before being rinsed in sterile water and dried. Then leaves were punched out and leaf discs were placed in sterile water. One hundred and twenty leaf discs (1.5 cm in diameter), divided into six groups for each fungus pathotype and control, were transferred into a sterilized petri dish. 5 mL of the fungus solution (1.0 × 10^7^ conidia per mL) was added to each petri dish. The control group was treated with a sterile medium. Inoculated leaf discs were cultured for 5 days at room temperature under a photoperiod of 10 h light and 14 h dark, and then the symptoms were observed, recorded, and photographed. The area of leaf damage was estimated with Image Pro Plus software (Image-Pro Plus Version 6.0, Media Cybernetics, Rockville, MD, USA). The leaf disc incidence ratio (diseased leaf disc/total leaf disc number) and disease speckle area ratio (the leaf disc area of yellow and brown/total leaf disc area) were calculated using ENVI 5.1 software. The data were analyzed with Prism 9 software.

Cotton seeds were sown in pots containing disinfested soil (nutrient soil: perlite: vermiculite, 3:1:1) and allowed to grow until the appearance of 2–3 leaves (30 days old) under greenhouse conditions. Each treatment and control group contained eighteen pots per cotton line with three plants (a total of 54 plants per cotton line in each group). Inoculums were harvested from one-week-old Czapek Dox liquid cultures, which were filtrated through four layers of gauze. The pathogenicity tests were performed by dipping the plant roots in a conidial suspension containing 1.0 × 10^7^ conidial spores per mL determined by a hemacytometer under a microscope (Olympus Corporation, Tokyo, Japan). Five *Verticillium* strains (three isolates and two reference strains) were inoculated to the treatment group separately using the root-dip method [10]. Un-inoculated control seedlings were dipped in sterile distilled water as the control group. The pots watered regularly were planted in an airconditioned greenhouse with a daily average temperature of 28–30 °C during the drought period (RH 30–35%) at a light regime of 16 h light and 8 h darkness. Disease symptoms appeared after 15 days of post-inoculation, and disease severity was recorded every 5 days until day 45. The verticillium symptoms were evaluated based on the ratio of wilted leaves in the whole plant. The disease symptoms were assessed from 0 to IV (0: healthy plant with no visible wilt symptoms; I: less than 25% chlorotic/necrotic leaves; II: between 25 and 50% chlorotic/necrotic leaves; III: between 50 and 75% chlorotic/necrotic leaves or defoliation; IV: more than 75% chlorotic/necrotic leaves or up to 90% defoliation; some terminal dieback, stunting, or death [30]. The disease index was calculated as follows:

$$DI=\frac{\sum \;(n\times disease\;grade\;of\;cotton\;plant\;N)}{4\times number\;of\;total\;seedlings}\times 100\%$$

where *n* denotes the number of seedling of each disease grade, N denotes the related disease grade of the cotton plant (0 to IV) [31]. Furthermore, chlorophyll content was tested after inoculating for 45 days using a SPAD-502 chlorophyll meter (Konica Minolta, Japan).

## 3. Data Analysis

All experiments were conducted with replications (more than five), and the SPSS software was used to analyze the data (Standard Version 16.0, Shanghai, China). All the values are expressed as the mean ± the standard error of the mean. The significance differences were determined by one-way analysis of variance (ANOVA) with Duncan and the differences with *p* values < 0.05 were considered statistically significant.

## 4. Results

### 4.1. Isolation of V. dahliae 

**Figure 1:**
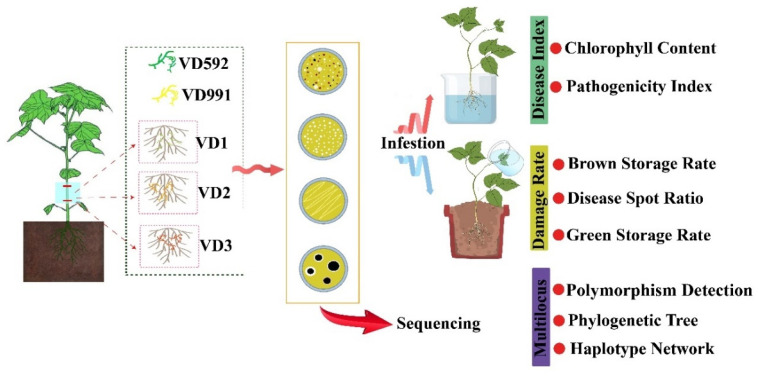
The simplified flowchart outlining the protocols for *V. dahliae* isolation and identification in this study.

To identify *V. dahliae* isolates and examine pathogenicity, molecular biological and microbiological techniques were performed under standard protocols. To this end, an exploratory strategy for stepwise identification of pathogenic isolates was established, as depicted in the graphical abstract (Figure 1).

**Figure 2:**
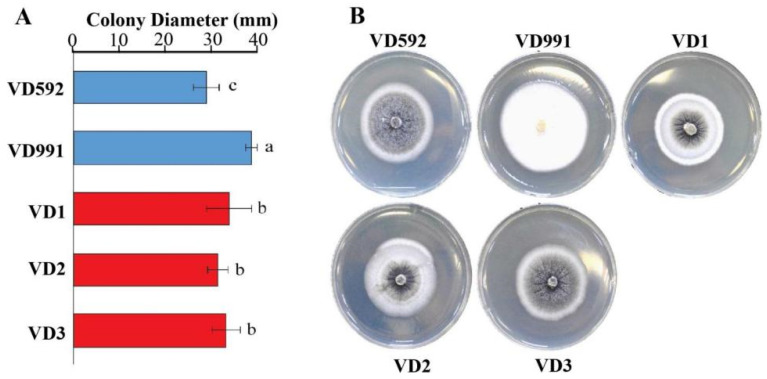
Cultural characterization of *V. dahliae* strains. (**A**) the colony diameter analysis after 10 days post culture. (**B**) the colony phenotype after 15 days of growth. The value represents the mean ± SE of six independent experiments. The letters indicate significant differences (*p* < 0.05) using one-way ANOVA following the Duncan test.

Fungal isolates from *Verticillium*-infected cotton stems were identified using morphological observation under a stereoscope (Supplemental Figure S1). The colony diameters of each fungus cultured on PDA medium for 10 days were measured, and statistical significance was tested by the Duncan method comparing it with reference strains. During the cultivation of the fungus, we discovered certain *V. dahliae* phenotypes that varied from one other. The results indicated that the growth rate of the reference VD991 strain was the fastest, followed by VD1, VD2, and VD3, whereas another reference strain VD592 had the slowest growth rate (Figure 2A). Based on morphological phenotype, these five strains can be divided into hyphae (VD991) and sclerotia (VD592, VD1, VD2, and VD3) types (Figure 2B). VD991 produced a large number of hyphae with white colonies on both the up- and downsides without microsclerotia. The other four strains (VD592, VD1, VD2, and VD3) produced microsclerotia in the middle of the colony, surrounded by white hyphae. However, VD1, VD2, and VD3 strains showed creases in the core of the colony, but VD592 had a rather smooth center.

### 4.2. Phylogenetic Analyses of ACT, EF, GPD, and TS Regions

**Figure 3:**
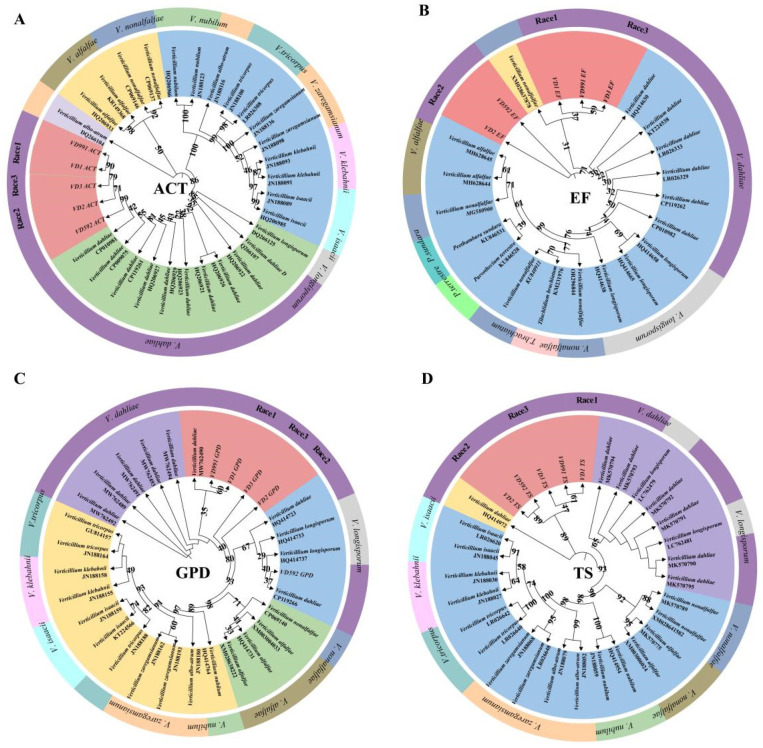
The ML tree based on four DNA barcodes. Bootstrap values greater than 22% indicate that a species was successfully identified. (**A**) The ML tree based on *ACT* sequences. (**B**) The ML tree based on *EF* sequences. (**C**) The ML tree based on *GPD* sequences. (**D**) The ML tree based on *TS* sequences.

Different morphological characteristics of fungal isolates indicated genetic variation. Herein, we further validated the isolates by the sequencing of the *ACT*, *GPD*, *EF*, and *TS* barcodes (Supplemental Figure S2). A BLAST analysis of sequences revealed 100% similarity with the publicly available *V. dahliae*. We compared the barcode sequences of the three *Verticillium* isolates with the referred pathogenic strains VD991 and VD592, as well as publicly available fungal barcodes in GenBank, to construct a phylogenetic tree (Figure 3). Results revealed that the majority of strains in this genus formed monophyletic branches with a high degree of similarity. The sequences extracted in this study in the phylogenetic tree based on the *GPD* loci showed well-formed monophyletic branches with *V. dahliae* strains. Overall, the above phylogenetic tree showed that the topology of the phylogenetic tree constructed by the *ACT* and *TS* loci was relatively stable (with a high confidence level) and could accurately distinguish the species of the genus (Figure 3).

### 4.3. Genetic Diversity and Pathotypes

**Figure 4:**
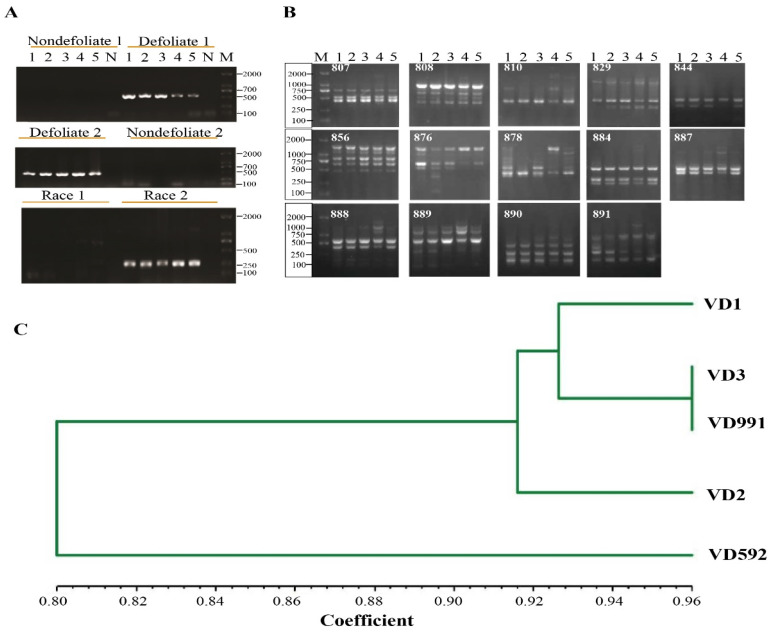
The molecular identity of cotton *V. dahliae* strains, the differentiated ISSR PCR productions and ISSR analysis. (**A**) 1–6 lines indicate VD592, VD991, VD1, VD2, and VD3 strains and a negative control; M, DL2000 marker. All primers information is shown in Table S1. (**B**) ISSR PCR productions: Lane M, DL2000 Marker, Lane 1 to 5 indicate PCR products from VD592, VD991, VD1, VD2, and VD3 strains. (**C**) Genetic correlation coefficient analysis using ISSR.

To determine fungal pathotypes and races among fungal isolates, relevant primer pairs were employed. Both defoliate-specific primer pairs D1/D2 and INTD2F/INTD2R produced about 550 bp and 462 bp bands, respectively, whereas non-defoliate-specific primers did not amplify any certain bands (Figure 4A). This demonstrated that fungal isolates from the cotton field belonged to a defoliate type of pathotype. Moreover, a race determination experiment revealed that all five strains were classified as race 2 and lacked the *Ave* gene (Figure 4A).

Inter-simple sequence repeats (ISSR) are considered as a rapid, reliable, and genome-wide covering approach for assessing genetic diversity. Therefore, we proceed with the fingerprint analysis using 29 ISSR primers to identify the three *V. dahlia* isolates, two reference strains. The band size ranged from 250 bp to 2000 bp (Figure 4B) and a total of 112 bands were obtained by amplification. The amplification strips were recorded and binary (0/1) matrices were constructed. NTsys2 software was used to analyze genetic similarity and plot the tree. The results indicated that five strains were divided into two large branches at a genetic similarity coefficient of 0.80, whereas VD592 was a separate branch, manifesting that they represent the different types of pathogenicity of *V. dahliae* strains (Figure 4C). Among these primers, 14 ISSR loci were present in diversity, the ISSR analysis distinguished the strains with similar characteristics, and VD2, VD1, and VD3 are largely divergent, indicating the genetic variation of the three strains (Figure 4C). The mycelial strain VD991 and the sclerotium type strain VD3 were clustered into one branch, and the genetic similarity coefficient was 0.96. The above results were consistent with the previous study, which demonstrated that different isolates of the same fungal species can generate profiles with polymorphic bands (Liu et al., 2023). 

### 4.4. Worldwide Haplotypes Analysis of V. dahliae

**Figure 5:**
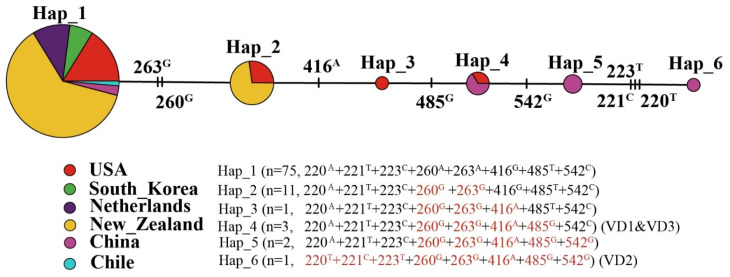
Distribution of fungal haplotypes based on tryptophan synthase (TS) sequence data. Geographic distribution of the haplotypes of *V. dahliae* strains populations and a Median-joining haplotype network of a *TS* fragment. Mutation sites are shown in red and conserved residues are shown in black.

Based on tryptophan synthase (TS) partial sequence data, the aligned sequences were normalized for sequence length by end trimming. The aligned sequences were 587 bp, with 8 bp variable sites and a nucleotide diversity of 0.3407. There were six distinct haplotypes in the present study. Notebly, the countries with the highest numbers of *V. dahliae* haplotypes were China (4) and the United States (4); the fewest numbers of haplotypes were found in the remaining countries (Figure 5). The most common haplotype globally was hap1 (ATCAAGTC) that estimated 85.23% (Figure 5). In addition, haplotype 4 is shared by China and the United States, notably including VD1 and VD3 fungal isolates. Haplotype 6 consisting of VD2 came from Xinjiang, China. Based on the mutation sites, this physiological race had three main variable sites (220T + 221C + 223T) compared with other homolog sequences.

### 4.5. Pathogenicity Determination of Fungal Isolates

**Figure 6:**
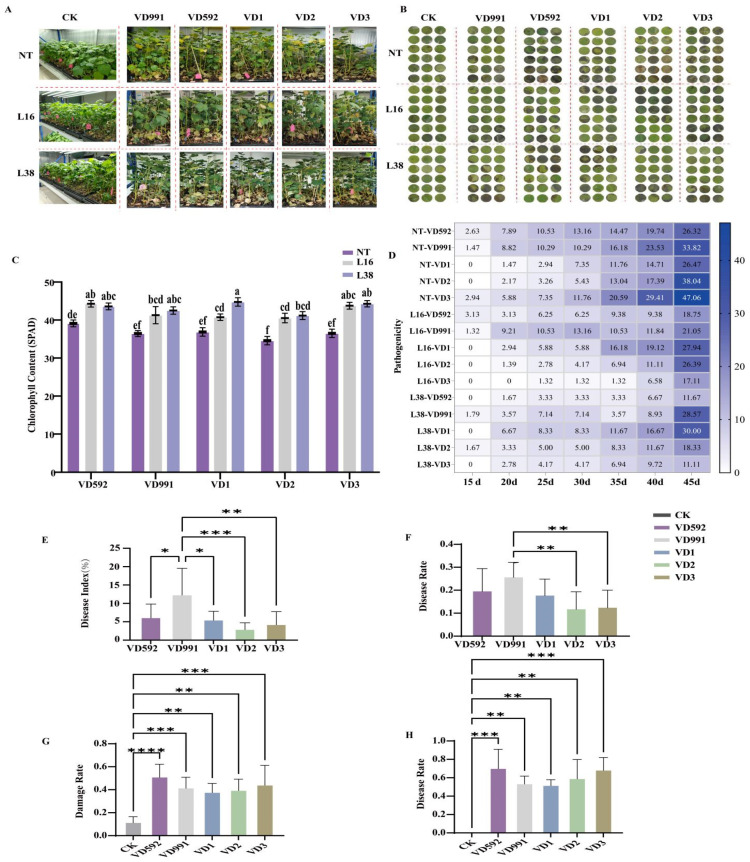
Pathogenicity of fungal isolates in plants. (**A**) The cottons infected with strains using the whole plant inoculation method. (**B**) The cottons infected with strains using the leaf disc inoculation method. (**C**) The chlorophyll content at 45 days post-inoculation. Each value represents the mean ± SE of three independent experiments. abcdef: Different lowercase letters denote significant differences between groups, *p* < 0.05. (**D**) The examination of distinct pathogenic strains for the whole cotton plant at various days following inoculation. (**E**) Disease index of the whole plant. (**F**) Damage ratio for the whole cotton plant. (**G**) Damage ratio of the leaves. (**H**) Disease ratio in the leaves. CK, not treated control. The values represent the mean ± SE of three independent experiments. Asterisks indicate significant differences (*p* < 0.05) according to one-way ANOVA following Duncan test. **** Significance: *p* < 0.0001, *** Significance: *p* < 0.001; ** Significance: *p* < 0.01; * Significance: *p* < 0.05.

The pathogenicity of *V. dahliae* pathotypes was thoroughly examined using leaf disc and whole plant inoculation methods to validate the pathogenicity level of fungal strains. For the whole plant inoculation, five *Verticillium* strains (two reference strains VD991, VD592, and three fungal isolates VD1, VD2, and VD3) were inoculated with the same spore concentration (10^7^ mL^−1^) into three-week-old seedlings of NT cotton plants, L16 and L38 (Table S2). After two weeks post-inoculation, wilting, necrosis, and vascular browning symptoms began (Figure 6A). As expected, different strains caused varying degrees of wilt symptoms on plants (Figure 6A). The VD991 strain especially damaged the cotton severely, whereas VD592, VD3, and VD1 strains had a lower impact, followed by VD2 to a lesser damage (Figure 6A). It is known that Verticillium wilt causes chlorophyll loss in leaves. After 45 days of inoculation, the results showed that the VD2 strain led to decreased chlorophyll content compared with the other four strains (VD991, VD592, VD1, and VD3), which resulted in similar chlorophyll losses (Figure 6C). Furthermore, the pairwise comparisons with different phases of pathogenicity revealed that these strains become more pathogenic with time, and the significant difference in pathogenicity is primarily shown in the middle stage (20–30 dpi), indicating that these strains aggravated the damage to plants significantly compared with that in the middle stage (Figure 6D). The disease index for the middle stage revealed that the pathogenicity of VD991 was very significant with VD2 and to a lesser extent with VD3, while there were the least significant differences between VD592 and VD1 (Figure 6E). In terms of disease rate, there were clear differences between VD991 and VD2 in the middle stages (Figure 6F), but no changes in disease rate were seen between strains at later stages.

For the leaf disc inoculation method, the disease damage was assessed from two perspectives: the area of leaf damage from the leaf disc incidence ratio and the disease speckle area ratio. Cotton leaf discs infected with *V. dahliae* isolates 5 days later revealed considerable damage to the cotton cultivar and its resistant transgenic lines (Figure 6B). After analyzing the leaf damage area, the disease damage ratio showed that the various strains had significantly varying pathogenicity (Figure 6B). Furthermore, the damage rate analysis results suggested that increased pathogenicity was seen for the reference VD592 strain, and significantly lesser pathogenicity for the VD1, VD2, VD3 isolates, and the reference V991 strain. (Figure 6G). Similarly, statistical analysis for the disease rate further confirmed the above findings for damage rate, with the very poor pathogenicity of VD1 (Figure 6H).

## 5. Discussion

*V. dahliae*, a root pathogen, causes destructive wilt disease in hundreds of plant species, severely limiting cotton production globally. Thus, it is of considerable scientific relevance to investigate the discrepancy and variety in the pathogenicity of *V. dahliae* strains. In this study, we isolated and characterized three *V. dahliae* species (VD1, VD2, and VD3) from cotton stems in cotton-growing bases in Manas County, Xinjiang-Uygur Autonomous Region, China, and compared their pathogenicity to two reference strains (VD592 and VD991) using different methods. The cultural and morphological variability of *V. dahliae* isolates evaluated in vitro demonstrated substantial variation in their mycelial growth, colony characteristics, colony features, and sclerotial formations. In the present study, VD991 (hyphae type) displayed a faster growing rate than other strains (clerotia type). We hypothesized that the three isolates had mildly pathogenic characteristics, but whether there was a difference in pathogenicity among the three strains was studied further in this research.

Five strains were tested by four pairs of specific primers (deciduous primers: D1/D2 and INTD2F/INTD2R, non-deciduous primers: ND1/ND2 and INNDDF/INTNDR), of which D1/D2 and ND1/ND2 were commonly used for identifying the pathogenic type of *V. dahliae* selected by RAPD technology [32]. The use of the two pairs of primers for complementary identification can increase the confidence level of the results. The previous reports revealed that strains from Xinjiang-Uygur Autonomous Region were dominated by non-deciduous types, but in recent years, the defoliating pathotype has dominated throughout China, and deciduous types often have more pathogenicity than non-deciduous ones [26,33]. Our results also indicated that *V. dahliae* isolates from cotton in Xinjiang belonged to the D pathotype and race 2, which was consistent with the report revealing that the D type correlated significantly with race 2, while the ND pathotype correlated with race 1 [34]. Because of the lack of the *Ave1* gene, race 2 type strains are less aggressive on plants. Ve1 was previously found in the study of tomatoes, lettuce, and sunflowers [19]. In recent years, the Ve1 gene was also found in cotton [35]. Based on these previous results, we hypothesize that some strains of *V. dahliae* may have a negative impact on cotton; strain-controlled and cotton-resistant breeding could be a solution for this.

Isoenzyme technology, RFLP, RAPD, and ISSR technology are commonly employed for determination of the genetic diversity of *V. dahliae* [26,32]. Previous studies found that the ACT, EF, GPD, and TS genes were suitable for species-level determination of *Verticillium* spp. [36]. However, ISSR technology was used to study the genetic diversity of *V. dahliae*. The ISSR data revealed that the genetic diversity of the three isolates was varied. The phylogenetic tree results based on the four loci showed that all five strains are always phylogenetically closely related to *V. dahliae* and varied from each other. Interestingly, the three isolates and reference strains formed a terminal branch that was distinct from other *V. dahliae* strains found from other countries, including within the same country. The substantial differences in phylogenetic connections suggested the presence of many cryptic physiologic races within the current isolated strains. Studies have demonstrated that the location of SNPs and Indels in the genome has an impact on gene expression and function [37], particularly the location of DNA polymorphisms in coding regions [38]. Thus, the result of TS located in the coding region revealed that the strains can be classified into six haplotypes, with the isolated strains differing from the globally found homologous strains. This may provide a crucial hint to pay close attention to variations in pathogenicity among the isolated strains. Then, three *V. dahliae* isolates, as well as the reference strains VD592 and VD991, were exposed to the root and leaf disc inoculation methods to remove pathogenicity inconsistencies. It was found that the three isolates, but with some varied pathogenicity from the above results, confirm the concept described previously. We found that *V. dahliae* isolates with existing genetic diversity belong to new physiologic races, and grow more slowly and less pathogenically than the reference strains based on a comprehensive pathogenicity assessment in the study. These findings also complement earlier research demonstrating that there was plasticity as a pathogenicity resulting from the genetic diversity in pathogen populations, and variability in co-evolutionary features with a host [3,4,16,30].

Our findings may offer insight on the evolutionary process of strains prevalent in the field, hence contributing to the research of pathogenicity and biological features of *V. dahliae* strains.

## 6. Conclusions

Overall, we discovered different *V. dahliae* strains in the same plant and isolated three *V. dahliae* physiologic races from the same cotton stem (VD1, VD2, and VD3). VD1, VD2, and VD3 are sclerotium types, like VD592 isolate. Moreover, VD991 is a mycelium pathogen, which is consistent with previous reports. Five strains are all defoliate-type pathogens. The isolated three strains were more genetically diverse, distinguished by ISSR and phylogenetic trees with ACT, EF, GPD, and TS loci. The result of TS with eight polymorphic loci revealed that the strains can be divided into six haplotypes globally, and the isolated strains belonged to two. Furthermore, pathogenicity results indicated that the isolated strains had the weakest pathogenicity compared to the reference strains (VD592 and VD991). In addition, the findings emphasize the importance of regular monitoring of pathogen species, as well as early detection of new, more virulent strains, to develop effective strategies for cotton-wilt management in practice.

## Data Availability

The original contributions presented in the study are included in the article/Supplementary Material, further inquiries can be directed to the corresponding authors.

## Supplementary Materials

The following supporting information can be downloaded at: https://www.mdpi.com/article/10.3390/pathogens13070525/s1, Figure S1. Six isolated fungi phenotype on media from cotton stems of Xinnongmian 1. Figure S2. The PCR barcoding results of isolated fungi. All primers information is shown in Table S1. The pathogenicity of five *V. dahliae* strains on three cotton lines at 45 days post-inoculation. Table S2 The pathogenicity of five *V. dahliae* strains on three cotton lines at 45 days post-inoculation.
