# Supplementary material for: Distribution and Pathogenicity Differentiation of Physiological Races of Verticillium dahliae from Cotton Stems in Western China
Source: Pathogens. 2024 Jun 21;13(7):525. doi: 10.3390/pathogens13070525 (PMC11280298; doi:10.3390/pathogens13070525)
Supplement: Supplementary file 1 [file pathogens-13-00525-s001.zip › pathogens-3055761-supplementary.pdf]

Distribution and pathogenicity differentiation of physiological races of *Verticillium dahliae* from cotton stems in Western China

Jianwei Zhang[1,2†], Aerguli Jiamahate [2†], Hui Feng[3], Tohir A. Bozorov[2,4], Dawei Zhang[5], Jianwei Guo[7], Honglan Yang[2]*, Daoyuan Zhang[2,6]*

Supporting information

Table S1 The primers used for molecular identification and fungal diversity in the study.

Used for	Function	Names	Sequences (5′-3′)
***Verticillium* strains identification**	TS	VTs5f	ACCTATGTCACTGCCGGCT
		VTs4r	CAATGAAGCCGTTGACGCC
	ACT	VActf	TAATTCACAATGGAGGGTAGG
		VActR	GTAAGGATACCACGCTTGG
	EF	VEFf	AACGTCGTCGTCATCGGCCACG
		VEFr	CCACGCTCACGCTCGGCCTT
	GPD	VGPDf2	GGCATCAACGGTTTCGGCC
		VGPDr	GTAGGAGTGGACGGTGGTCATGAG
**Pathotypes**	Defoliate 1	D1	CATGTTGCTCTGTTGACTGG
		D2	GACACGGTATCTTTGCTGAA
	Defoliate 2	INTND2F	ACTGGGTATGGATGGCTTTCAGGACT
		INTND2R	TCTCGACTATTGGAAAATCCAGCGAC
	Non-Defoliate 1	ND1	CAGGGGATACTGGTACGAGACG
		ND2	ATGAGTATTGCCGATAAGAACA
	Non-Defoliate 2	INTNNDF	CCACCCCAAGCGACAAGAC
		INTNNDR	TAAAACTCCTTGGGGCCAGC
**Race type**	race1	VdAve1F	AAGGGGTCTTGCTAGGATGG
		VdAve1R	TGAAACACTTGTCCTCTTGCT
	race2	VdR2F	ACTTAACGAAAGCATGCGC
		VdR2R	CTTGACTTGCCGGCTCC
**Genetic diversity**	ISSR	807	AGAGAGAGAGAGAGAGT
		808	AGAGAGAGAGAGAGAGC
		810	GAGAGAGAGAGAGAGAT
		811	GAGAGAGAGAGAGAGAC
		815	CTCTCTCTCTCTCTCTG
		816	CACACACACACACACAT
		826	ACACACACACACACACC
		827	ACACACACACACACACG
		829	TGTGTGTGTGTGTGTGC
		836	AGAGAGAGAGAGAGAGYA
		840	GAGAGAGAGAGAGAGAYT
		844	CTCTCTCTCTCTCTCTRC

	850	GTGTGTGTGTGTGTGTYC
	855	ACACACACACACACACYT
	856	ACACACACACACACACYA
	858	TGTGTGTGTGTGTGTGRT
	859	TGTGTGTGTGTGTGTGRC
	866	CTCCTCCTCCTCCTCCTC
	873	GACAGACAGACAGACA
	876	GATAGATAGACAGACA
	878	GGATGGATGGATGGAT
	884	HBHAGAGAGAGAGAGAG
	885	BHBGAGAGAGAGAGAGA
	886	VDVCTCTCTCTCTCTCT
	887	DVDTCTCTCTCTCTCTC
	888	BDBCACACACACACACA
	889	DBDACACACACACACAC
	890	VHVGTGTGTGTGTGTGT
	891	HVHTGTGTGTGTGTGTG

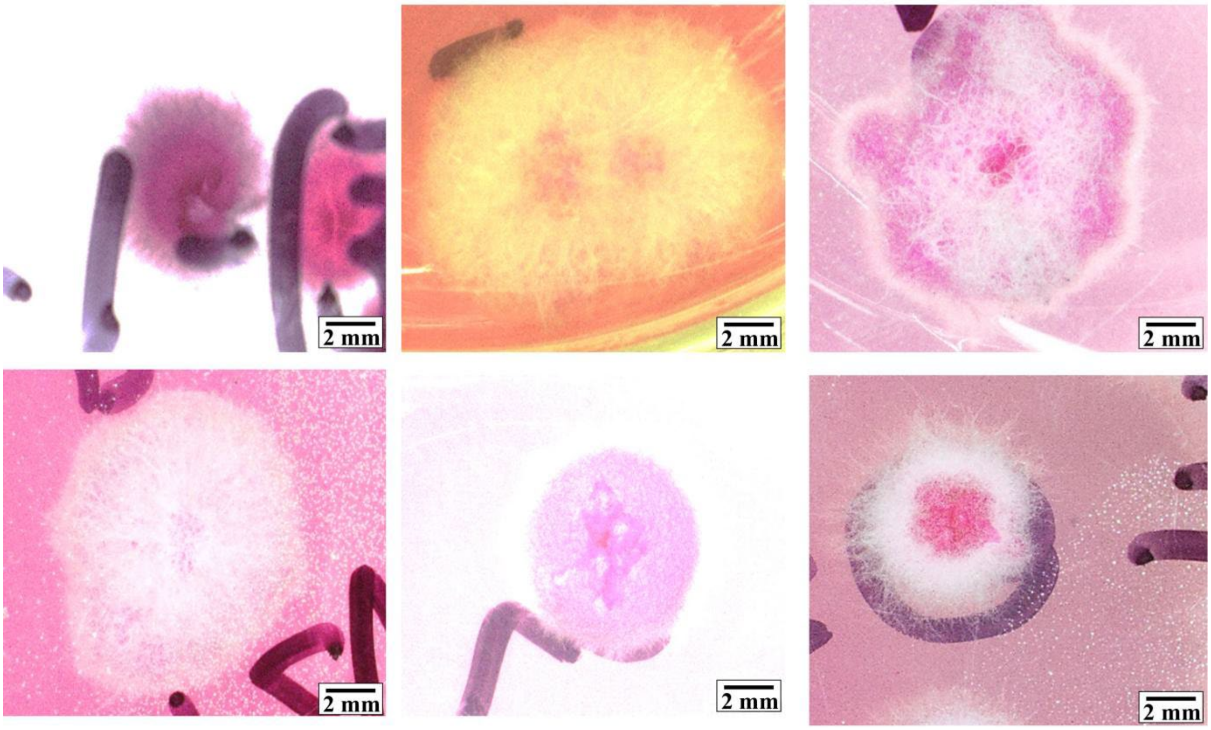

Figure S1 Six isolated fungi phenotype on media from cotton stems of Xinnongmian 1.

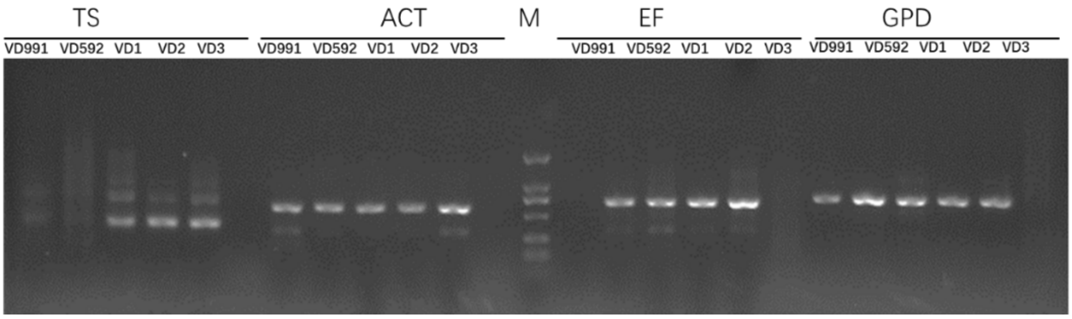

Figure S2 The PCR barcoding results of isolated fungi. All primers information is shown in Table 1.

Table S2 The pathogenicity of five *V. dahliae* strains on three cotton lines at 45 days post-inoculation.

Strains	NT		L16		L38	
	DI	RT	DI	RT	DI	RT
V592	26.32	T	18.75	R	11.67	R
V991	33.82	T	21.05	R	28.57	T
VD1	26.47	T	27.94	T	30.00	T
VD2	38.04	T	26.39	T	18.33	R
VD3	47.06	T	17.11	R	11.11	R

Notes: DI, disease index; RT, reaction type to pathogen；R, resistance; T, tolerance. NT, non-transgenic cotton; L16 and L38, transgenic *ScALDH21* cotton lines.
